# Prospective hospital-based case–control study to assess the effectiveness of pandemic influenza A(H1N1)pdm09 vaccination and risk factors for hospitalization in 2009–2010 using matched hospital and test-negative controls

**DOI:** 10.1186/1471-2334-12-127

**Published:** 2012-05-31

**Authors:** Wiebke Hellenbrand, Pernille Jorgensen, Brunhilde Schweiger, Gerhard Falkenhorst, Matthias Nachtnebel, Benedikt Greutélaers, Christian Traeder, Ole Wichmann

**Affiliations:** 1 Immunization Unit, Department of Infectious Disease Epidemiology, Robert Koch Institute, DGZ-Ring 1, 13086 Berlin, Germany; 2 National Reference Centre for Influenza, Robert Koch Institute, Berlin, Germany; 3 Post Graduate Training in Applied Epidemiology, Robert Koch Institute, Berlin, Germany; 4 Department of Infectious Diseases, Vivantes Auguste-Viktoria Klinik, Berlin, Germany

**Keywords:** Influenza, H1N1, Vaccine effectiveness, Case–control study, Hospitalization, Risk factors

## Abstract

**Background:**

We performed a case–control study to estimate vaccine effectiveness (VE) for prevention of hospitalization due to pandemic influenza A(H1N1)pdm09 (pH1N1) and to identify risk factors for pH1N1 and acute respiratory infection (ARI) in 10 hospitals in Berlin from December 2009 to April 2010.

**Methods:**

Cases were patients aged 18–65 years with onset of ARI ≤10 days before admission testing positive for pH1N1 by PCR performed on nasal and throat swabs or by serological testing. Cases were compared to (1) matched hospital controls with acute surgical, traumatological or other diagnoses matched on age, sex and vaccination probability, and (2) ARI patients testing negative for pH1N1. Additionally, ARI cases were compared to matched hospital controls. A standardized interview and chart review elicited demographic and clinical data as well as potential risk factors for pH1N1/ARI. VE was estimated by 1-(Odds ratio) for pH1N1-vaccination ≥10 days before symptom onset using exact logistic regression analysis.

**Results:**

Of 177 ARI cases recruited, 27 tested pH1N1 positive. A monovalent AS03-adjuvanted pH1N1 vaccine was the only pandemic vaccine type identified among cases and controls (vaccination coverage in control group 1 and 2: 15% and 5.9%). The only breakthrough infections were observed in 2 of 3 vaccinated HIV positive pH1N1 patients. After exclusion of HIV positive participants, VE was 96% (95%CI: 26-100%) in the matched multivariate analysis and 46% (95%CI: -376-100%) in the test-negative analysis. Exposure to children in the household was independently associated with hospitalization for pH1N1 and ARI.

**Conclusions:**

Though limited by low vaccination coverage and number of pH1N1 cases, our results suggest a protective effect of the AS03-adjuvanted pH1N1 vaccine for the prevention of pH1N1 hospitalization. The use of hospital but not test-negative controls showed a statistically protective effect of pH1N1-vaccination and permitted the integrated assessment of risk factors for pH1N1-infection. To increase statistical power and to permit stratified analyses (e.g. VE for specific risk groups), the authors suggest pooling of future studies assessing effectiveness of influenza vaccines for prevention of severe disease from different centres.

## Background

In June 2009 the World Health Organization (WHO) declared a global pandemic after the emergence of a novel influenza A(H1N1) virus initially in Mexico and the USA that rapidly spread world-wide [1,2]. In Germany, vaccination against pandemic influenza A(H1N1)pdm09 (from here on referred to as pH1N1) was initiated 2 weeks into the main pandemic wave in calendar week 44 [3] with Pandemrix®, a monovalent AS03-adjuvanted pH1N1-vaccine produced by GlaxoSmithKline. A non-adjuvanted vaccine was introduced 7 weeks later, but was restricted to pregnant women. Preliminary results of a randomized clinical trial of the AS03-adjuvanted vaccine containing 5.25 μg haemagglutinin showed seroconversion and seroprotection rates >96% after one dose [4]. Later results confirmed excellent immunogenicity of the widely implemented 3.75 μg dose formulation in adults up to age 85 years [5].

While immunogenicity data remain the basis for licensure of influenza vaccines, correlation with clinical protection against pandemic influenza after pH1N1-vaccination was not known. Therefore, we launched a hospital-based case‐control study in Berlin to estimate the effectiveness of pH1N1-vaccination for prevention of hospitalization due to pH1N1. Secondary objectives were to determine the effect of previous vaccination with seasonal influenza vaccine on the risk of hospitalization with pH1N1, and to identify risk factors for hospitalization with pH1N1 or acute respiratory infection (ARI).

## Methods

### Study design and setting

We performed a prospective hospital-based case–control study in nine community hospitals comprising the Vivantes Network for Health located in seven of the 12 Berlin districts and one of the Charité University hospitals located in an eighth district, together serving approximately one third of the Berlin population of 3.8 million inhabitants from week 50, 2009 to week 14, 2010.

### Study population

ARI cases were defined as patients aged 18–65 years admitted to the participating hospitals with symptom onset ≤10 days prior to admission and the following clinical criteria for ARI: [(fever >38.0°C or other systemic symptoms such as chills, headache, myalgia/arthralgia) AND (cough or sore throat)] OR [influenza-like illness, influenza, acute bronchitis or pneumonia suspected/diagnosed by a physician]. Older patients were not recruited, since a low pH1N1-incidence was expected among them based on the age distribution of cases notified in Germany at the time the study was planned (<1% of cases aged >60 years [6]). Newly admitted ARI patients were identified by daily screening of the hospital information system. We excluded patients unable to give informed consent or to communicate adequately, those with an absolute contra-indication for influenza vaccination (history of anaphylaxis from vaccine components) or with a history of prior laboratory-confirmed pH1N1-infection.

As one of the centres specialized in the treatment of HIV patients, we expected to recruit a disproportionately high number of HIV-positive participants. Rather than exclude HIV patients from recruitment, we decided to exclude them from the primary VE-analysis, but to undertake a separate VE analysis among HIV patients if a sufficient number could be recruited..

Newly admitted patients with either acute surgical, traumatological, urological, nephrological, gynaecological or medical diagnoses or with minor elective surgical procedures were also documented daily as potential controls. For each case patient, a control was then randomly selected and recruited according to the following matching criteria: date of admission (+/− 10 days), age (+/− 10 years), sex and vaccination probability. Medical personnel, persons with underlying chronic illness, pregnant women and police/fire workers were defined as having a high vaccination probability based on the official vaccination recommendations that prioritized these groups for receipt of pH1N1-vaccine [7,8]. Exclusion criteria for controls were as described for cases along with a history of a respiratory illness with sudden onset and fever and cough and either myalgia, arthralgia or headache since October 1, 2009, OR serological evidence of previous pH1N1-infection.

### Sample size calculation

We assumed a moderate to high vaccine effectiveness (VE) based on the literature on seasonal influenza vaccination in healthy adults with a good vaccine/circulating virus match [9,10]. We expected low to moderate vaccination coverage in view of controversial public discussion on pandemic vaccination in Germany at the time [11]. Thus, at a power of 80% and with 2 controls per case, we estimated we would need to recruit 85 (VE = 90%; vaccination coverage = 30%) to 249 pH1N1 positive cases (VE = 60%; vaccination coverage = 10%).

### Data collection

A standardized interview elicited demographic data, illness onset, clinical symptoms, vaccination history and risk factors for respiratory infections or potential confounders such as demographic factors, underlying illness, smoking, body mass index (BMI), functional health status, contact with children, use of public transport and car ownership. When possible, the vaccination history was obtained from the vaccination record; if unavailable, it was requested from the family doctor. If neither approach was possible, we documented the data from the patient’s memory; however, if a reportedly vaccinated patient was unable to recall at least the month/year of pH1N1 or seasonal 2009/2010 influenza vaccination or the year of pneumococcal vaccination, vaccination status was considered unknown. For seasonal 2007/2008 or 2008/2009 vaccination, recall of the year of vaccination was considered sufficient. The patient’s chart was reviewed after discharge to retrieve clinical and laboratory data on illness severity/progression, underlying illness, as well as radiological and microbiological findings.

The patient’s own or a report in the medical record was considered sufficient evidence for underlying medical conditions. We defined chronic lung disease as at least one of chronic obstructive lung disease (COPD), asthma, chronic bronchitis or regular use of bronchodilators, and underlying cardiovascular disease as a history of heart disease, stroke or use of antiplatelet agents, but not isolated hypertension. “Frailty” was defined as requiring help with personal hygiene, with walking or with meals. Regular use of public transport was defined as use at least 3 times per week. The status of either having or being eligible for private health insurance was used as a proxy for higher income. We defined high educational status as having completed at least grade 10, required for further vocational training in Germany.

### Laboratory methods

Nasal and throat swabs (Mastaswab; MAST Diagnostica, Reinfeld, Germany) were collected from consenting ARI cases, stored refrigerated and shipped to the National Reference Centre for Influenza (NIC) within 1–4 days. If patients were intubated, tracheal secretions (n = 1) or bronchioalveolar lavage fluid (n = 6) were tested. Samples were washed out in 1.5 ml cell culture medium and combined. RNA was extracted from 300 μl of the pooled sample using the MagAttract Viral RNA 48 Kit (Qiagen) and eluted in 80 μl elution buffer. cDNA was synthesized using 25 μl of RNA and 200U M-MLV Reverse Transcriptase (Invitrogen) in a total reaction volume of 40 μl. Real-time polymerase chain reaction (PCR) was performed as described previously, targeting the M gene for universal detection of influenza A viruses as well as the HA and NA genes for further subtyping including the specific detection of pH1N1 viruses [12]. Identification and differentiation of influenza B viruses were performed according to Biere et al. [13].

A serum sample was requested from ARI cases and hospital controls upon recruitment, and from cases again 2–4 weeks later. Haemagglutination inhibition (HI) was performed at NIC to measure pH1N1-specific antibodies by as described previously [14], using the reference strain A/California/7/2009. An international pH1N1 serum standard (HI-titre: 1:183) provided by the National Institute for Biological Standards and Control, London, UK, was included in addition to negative and positive human control sera. A comparable titre of 1:160 was obtained for the standard in different runs. HI titres were expressed as the reciprocal of the last serum dilution preventing haemagglutination. The minimum detection limit was 1:10.

### Data analysis

Patients were considered pH1N1-positive if the nasopharyngeal swab was PCR-positive or if pH1N1 serum antibody-titres increased either from 0 to ≥40 or at least 4-fold in the acute and convalescent sera. Patients were considered pH1N1-negative if a nasopharyngeal swab taken within 7 days of symptom onset was PCR-negative. Patients with PCR-negative swabs taken >7 days after symptom onset were considered pH1N1-negative only if the pH1N1 antibody titre in both serum samples was 0, or - if pH1N1-vaccinated - if the titre remained stable (≤ 2-fold increase) or decreased. The cut-off at day 7 was chosen based on several studies investigating shedding of influenza viruses that showed a low probability of PCR-positivity after this time [15-17], although two studies in elderly hospitalized patients showed >50% of patients were still shedding virus 7 days after symptom onset [18,19]. We performed a sensitivity analysis using a cut-off of 4 days. Recruited controls with serological evidence of previous pH1N1-infection (HI titer ≥ 40) were excluded from the analyses.

The study period was divided into periods 1 (week 48, 2009 to week 2, 2010) with high-level, and 2 (week 3 to week 17, 2010) with low-level pH1N1 circulation [20].

**Figure 1:**
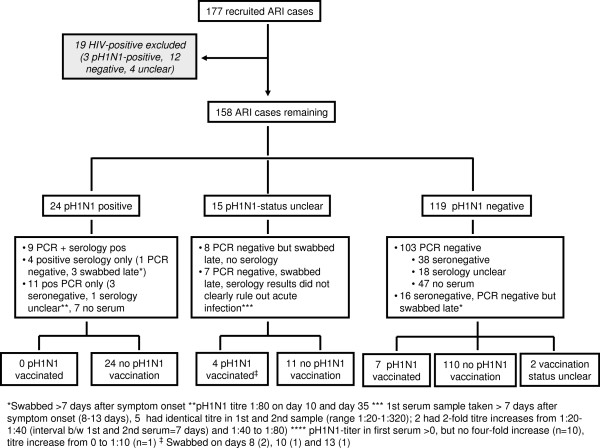
Recruited ARI patients according to pH1N1 diagnosis and pH1N1-vaccination status.

Patients were excluded from VE analysis if they were HIV-positive or if their pH1N1-status or vaccination status could not be determined (see Figure 1). Since studies showed high VE [21] and immunogenicity [22] for the AS03-adjuvanted pH1N1-vaccine already 7 days after vaccination, as well as similar VE 7–10 days after vaccination as after the more conventional 14 days [23-27], we considered ARI patients and hospital controls pH1N1-vaccinated if they received ≥1 dose of pandemic vaccine ≥10 days prior to symptom onset and admission, respectively. We performed sensitivity analyses using a 14-day cut-off.

VE, calculated as [(1-Odds ratio (OR)) x 100%] and risk factors for hospitalization with pH1N1 were determined in the following comparisons: (1) pH1N1-positive ARI cases compared to matched hospital controls using exact logistic regression to calculate matched odds ratios (MOR) with the *group* function in STATA (Version 11.0, STATA Corp.); (2) pH1N1-positive ARI cases compared to pH1N1-negative ARI-patients as controls using multivariate exact logistic regression in SAS (version 0.2, SAS Institute Inc.).

To identify risk factors for hospitalization with ARI, ARI cases were compared to matched hospital controls using conditional logistic regression in STATA. Proportions were compared using the chi-square test.

### Informed consent and ethical approval

All participants provided written informed consent. The study was approved by the local Ethics Committee of Charité University Medicine Berlin.

## Results

### Study participants

Of 299 identified ARI patients 177 (59%) consented to participate in the study. Recruited and non-recruited patients did not differ with respect to age and sex distribution, nor regarding the frequency of key symptoms such as fever, cough/sore throat or dyspnoea. The proportion of ARI cases testing pH1N1-positive was higher in period 1 (22/63, 35%) than period 2 (5/114; 4%; p < 0.001). Among ARI patients aged <55 years, 19.2% tested pH1N1-positive versus 7.4% among 55–65 year-olds (p = 0.05). Figure 1 describes the ARI cases according to their laboratory results and pH1N1-vaccination status; 19 HIV-positive ARI cases (8 vaccinated) were excluded from VE analyses. Two ARI cases (including one pH1N1-positive) were pregnant.

**Figure 2:**
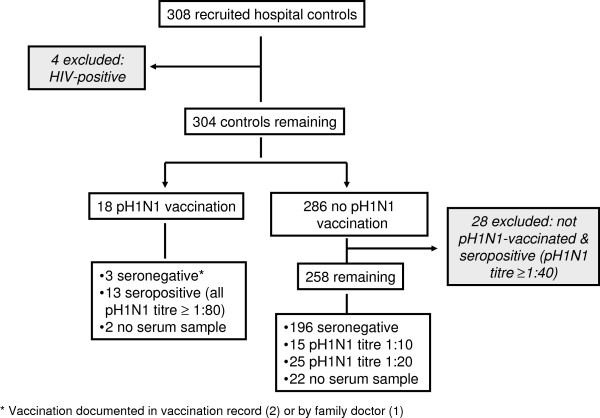
Recruited hospital controls according to pH1N1 diagnosis and pH1N1-vaccination status.

We recruited 308 hospital controls (Figure 2), of which 4 HIV patients (3 pH1N1-vaccinated) were excluded. Of 304 remaining controls, 28 (9.8%, all unvaccinated) were excluded because of a pH1N1-specific HI titre ≥1:40. Of the remaining 276 controls, 55% were acute surgical patients, 35% had planned surgical interventions and 10% other acute diagnoses.

### Vaccination status

The only pH1N1-vaccination breakthrough infections occurred in 2/3 pH1N1-positive HIV-patients; none of the remaining 24 pH1N1-positive cases were pH1N1-vaccinated. Overall, 11/158 (7.0%) recruited ARI cases and 18/276 (6.5%) hospital controls were pH1N1-vaccinated (Figures 1 and 2), compared to 30.4% and 22.2% vaccinated with seasonal 2009/2010 influenza vaccine, respectively. pH1N1-vaccination coverage was similar in study participants with high and low vaccination probability (7.1% (20/281) versus 5.0% (9/179), p = 0.37). All vaccinated participants received one dose of Pandemrix® 12–120 days before symptom onset (ARI cases) or hospital admission (hospital controls) with the exception of 1 hospital control with 2 doses 34 and 13 days before admission.

Both pH1N1-vaccination and 2009/2010 seasonal influenza vaccination status were documented in the vaccination record or confirmed by the vaccinating physician in a high proportion of vaccinated (90%), but in a low proportion of unvaccinated (25%) study participants and this was similar in cases and controls included in the VE analyses (Table 1).

**Table 1:** Proportion of participants with documented vaccination status (vaccination record or family physician) according to reported vaccination status and study group

	**pH1N1 vaccinated**	**pH1N1 unvaccinated**	**Seasonal influenza 09/10 vaccinated**	**Seasonal influenza 09/10 unvaccinated**
	Proportion with documented vaccination status* (%)			
**pH1N1 positive ARI cases (N = 24)**	0/0	7/24 (29%)	6/6 (100%)	5/18 (28%)
**Hospital controls matched to pH1N1 positive cases (N = 40)**	6/6 (100%)	12/34 (35%)	7/9 (78%)	10/39 (33%)
**pH1N1 negative ARI cases (test negative controls, N = 119)**	6/7 (86%)	30/110 (27%)	31/36 (86%)	19/81 (23%)
**HIV patients**	9/10 (90%)	11/12 (92%)	13/14 (93%)	7/7 (100%)

*Number of participants with documented vaccination status/total number of study participants in group (%).

### Comparison of pH1N1-positive cases with matched hospital controls

Because the only control of one case had to be excluded due to seropositivity, 23 pH1N1-positive cases (mean age: 41.5 ± 14.1 years, 74% male) were compared to 40 matched controls (41.5 ± 12.7 years, 73% male); 6 were paired with 1 control and 17 with 2 controls. Of the 40 controls, 6 (15.0%) were pH1N1-vaccinated.

As none of the pH1N1-positive cases was vaccinated against pH1N1, the crude MOR and VE estimates were 0 and 100%, respectively. The univariate median unbiased estimate of the MOR calculated using exact logistic regression analysis for pH1N1-vaccination was 0.19 (95%CI: 0–1.54, p = 0.13), corresponding to a VE of 81% (95% CI: -54%-100%). A number of other factors were associated with pH1N1-infection in exact logistic regression analysis (Table 2), although only exposure to children in the household (MOR: 4.3, 95% CI: 1.0-26.0, p = 0.04) and ≥1 hospitalization in the past year (MOR = 0.2, 95% CI: 0–0.7, p = 0.01) reached statistical significance. A positive association with pH1N1 illness that approached statistical significance was observed for COPD (p = 0.06), immunosuppression (p = 0.07), BMI (p = 0.06), car ownership (p=0.06) and a negative association for having been offered pandemic vaccine by one’s employer (p = 0.07) and use of public transport (p = 0.06, see Table 2). None of the variables associated with pH1N1 illness was significantly associated with pH1N1-vaccination in this data set (results not shown). However, among controls (but not cases), owning a car was positively (p = 0.05) and frequent use of public transport inversely (p = 0.06) associated with children in the household, possibly explaining the positive association between car ownership (p = 0.06) and pH1N1 illness. There was no association between pH1N1 illness and seasonal influenza vaccination, smoking, educational status or health insurance status (Table 2).

**Table 2:** Comparison of pH1N1-positive cases with matched hospital controls and test-negative controls, respectively: Analysis of vaccination status, underlying illness and other potential risk factors

	***pH1N1 positive cases*vs.*matched hospital controls***						***pH1N1 positive cases*vs.*pH1N1 negative controls***					
**Variable**	**Cases (23)**		**Controls (40)**		MOR* (95% CI)	***p***	**Cases (24)**		**Controls (119)**		***Exact OR*** (95% CI)**	***p***
	***N***	***%***	***N***	***%***			***N***	***%***	***N***			
**Pandemic influenza vaccination 2009/10**	*23*	0	*40*	15	0.2 (0–1.5)	0.13	*24*	0	*117*	6	0.5 (0–4.8)	0.62
**Seasonal influenza vaccination 2009/10**	*23*	26	*39*	23	1.3 (0.2-7.5)	0.99	*24*	25	*117*	31	0.8 (0.2-2.8)	0.95
**Seasonal influenza vaccination 2008/09**	*23*	26	*40*	25	1.1 (0.3-4.1)	1.0	*24*	25	*111*	19	3.4 (0.6-20.8)	0.21
**Seasonal influenza vaccination 2007/08**	*23*	22	*40*	20	1.2 (0.2-7.6)	1.00	*24*	21	*110*	16	2.4 (0.4-14.9)	0.45
**Pneumococcal vaccine**	*20*	20	*35*	11	2.3 (0.4-15.9)	0.49	*21*	24	*103*	13	3.4 (0.6-19.5)	0.19
**Pandemic vaccine offered by employer**	*23*	9	*40*	30	0.1 (0–1.1)	0.07	*24*	8	*115*	12	1.1 (0.1-6.7)	1.00
**Any chronic underlying illness**	*23*	70	*40*	63	1.7 (0.1-117.7)	1.0	*24*	67	*119*	66	1.5 (0.4-6.3)	0.72
**Any chronic lung disease**	*23*	43	*40*	23	2.2 (0.6-8.6)	0.27	*24*	42	*118*	34	2.2 (0.6-7.9)	0.25
**COPD**	*23*	22	*40*	3	7.9 (1.0-∞)	0.06	*24*	21	*118*	27	1.2 (0.3-5.2)	0.99
**Asthma**	*23*	26	*40*	15	1.8 (0.4-8.9)	0.58	*24*	25	*118*	16	1.4 (0.3-5.5)	0.77
**Chronic bronchitis**	*23*	13	*40*	5	3.2 (0.2-177.0)	0.61	*24*	13	*118*	17	0.9 (0.1-4.5)	1.00
**Cardiovascular disease**	*23*	39	*40*	28	2.6 (0.4-29.2)	0.44	*24*	38	*119*	40	1.4 (0.3-5.6)	0.84
**Immunosuppression**	*21*	14	*40*	0	7.7 (0.8-∞)	0.07	*22*	14	*116*	15	1.1 (0.2-5.5)	1.00
**BMI ≥31**	*23*	30	*39*	8	5.1 (0.9-51.2)	0.06	*24*	29	*118*	17	2.3 (0.6-9.1)	0.27
**Frailty**	*23*	4	*40*	3	1.4 (0–117.7)	1.00	*24*	4	*118*	9	0.4 (0.1-3.4)	0.65
**≥ 1 physician visits in past year**	*23*	70	*39*	72^†^	1.0 (0.3-3.3)	1.00	*24*	*67*	*118*	73^†^	1.7 (0.5-6.6)	0.54
**≥ 3 physician visits in past year**	*23*	48	*39*	38^‡^	1.5 (0.4-4.9)	0.64	*24*	*46*	*118*	53^‡^	1.3 (0.4-4.2)	0.84
**≥ 1 hospitalization in past year**	*23*	17	*39*	49	0.2 (0–0.7)	0.01	*24*	17	*119*	32	0.4 (0.1-1.6)	0.26
**Current smoker**	*23*	61	*40*	53	1.3 (0.4-3.8)	0.81	*24*	58	*119*	40	1.6 (0.5-4.8)	0.53
**>20 packyears vs. non-smoker**	*23*	30	*40*	25	1.8 (0.4-11.1)	0.69	*24*	29	*119*	33	2.2 (0.4-14.4)	0.52
**Alcohol: ≥ 1 drink/week**	*23*	43	*40*	65	0.3 (0.1-1.1)	0.08	*24*	45	*117*	56	0.6 (0.2-1.8)	0.40
**Children in household**	*23*	39	*40*	15	4.3 (1.0-26.0)	0.04	*24*	38	*119*	36	1.0 (0.3-3.0)	1.00
**Public transport (> = 3x/week) vs. none**	*23*	30	*40*	58	0.2 (0–1.1)	0.06	*24*	29	*119*	45	0.3 (0.1-1.0)	0.06
**Owns car**	*23*	74	*40*	45	3.3 (1.0-14.3)	0.06	*24*	75	*119*	51	3.6 (1.1-13.9)	0.04
**Completion of at least Grade 10**	*23*	68	*40*	77	0.6 (0.1-3.1)	0.72	*24*	71	*117*	69	1.5 (0.5-5.4)	0.66
**Private health insurance**	*23*	24	*40*	13	2.1 (0.2-26.2)	0.74	*24*	25	*119*	17	1.2 (0.3-4.2)	1.00

*MOR calculated with exact logistic regression. **Adjusted for time period and age.

Comparison of hospital and test-negative controls: †pχ2 = 0.11 ‡pχ2 = 0.90.

In multivariate analysis, the MOR for pH1N1-vaccination was 0.04 (95%CI: 0–0.74), corresponding to a VE of 96% (95%CI: 26-100%). In the final model, pH1N1-vaccination remained predictive of pH1N1 illness, together with the variables “contact to children in the household” (OR = 9.2, 95%CI: 1.1–440.0, p = 0.03) and “≥1 hospital admission in previous year” (OR = 0.1, 95%CI: 0–0.6, p = 0.01). The OR for additional variables added to the model did not reach statistical significance and had minimal further influence on the effect of these covariates. If only persons vaccinated ≤14 rather than ≤10 days prior to symptom onset (ARI cases) or hospitalization (hospital controls) were considered to be pH1N1-vaccinated, the estimated OR for pH1N1-vaccination changed only minimally (univariate estimate: 0.20, 95%CI: 0–1.87; multivariate estimate: 0.04, 95%CI: 0–0.69).

### Comparison of pH1N1-positive cases with test-negative controls

In the test-negative analysis, 24 pH1N1-positive ARI cases were compared to 119 pH1N1-negative ARI cases (Figure 1), of whom 7 (5.9%) were vaccinated, all >21 days prior to symptom onset. pH1N1-positive cases were significantly younger (41.6 ± 12.5 years) than test-negative cases (48.1 ± 12.3 years, p = 0.02) and more likely to have had symptom onset in time period 1 (83% versus 28%, p < 0.0001). Therefore, all analyses were adjusted for age and time period. The sex distribution (71% vs. 61% men, p = 0.11) and the proportion of patients with a higher vaccination probability (67% vs. 59%, p = 0.28) were similar.

The OR for pH1N1-vaccination was 0.54 (95% CI: 0–4.76), yielding a VE of 46% (95%CI: -376%–100%). We did not find a significant association between pH1N1 illness and seasonal influenza vaccination, although OR estimates for the 2008/2009 and the 2007/2008 seasonal vaccination were non-significantly increased. Significant associations between further variables and pH1N1-positivity were not found in this analysis (Table 2), with the exception of car ownership (OR = 3.6, 95%CI: 1.1–13.9, p = 0.04). The inverse association between pH1N1-positivity and public transport also seen in the matched analysis approached statistical significance (p = 0.06). Among controls (but not cases), owning a car was positively (p = 0.07) and frequent use of public transport inversely (p = 0.17) associated with children in the household, but exposure to children was similar in cases and test-negative controls in this analysis (Table 2). Addition of variables with an OR having p < 0.20 singly to the exact logistic regression equation containing age, time period and pH1N1-vaccination had minimal effect on the OR for pH1N1-vaccination.

When using a cut-off of 4 rather than 7 days after symptom onset as the interval for considering a negative PCR result as definitively negative, the OR for pH1N1-vaccination changed minimally (OR = 0.50 (95%CI: 0–4.44), equivalent to a VE of 50% (95%CI: -344–100%)).

### Factors associated with hospitalization for ARI

After exclusion of HIV positive ARI-cases and hospital controls, 6 ARI-cases were left without a hospital control, and 30 hospital controls no longer had a case. Thus, 152 ARI-cases (mean age: 47.0 ± 12.7 years, 64% male) were compared to 246 matched hospital controls (47.6 ± 12.5, 65% male); 58 cases were paired with one and 94 with 2 controls. Results of the univariate conditional logistic regression analysis are shown in Table 3. In multivariate backwards conditional logistic regression, only COPD (OR = 19.4, 95%CI: 4.4-85.8, p < 0.0001), exposure to children in the household (OR = 2.4, 95%CI: 1.3-4.5, p = <0.007), and having had pH1N1-vaccine offered by an employer (OR = 0.4, 95%CI: 0.2-0.8, p = 0.02) remained independently and significantly associated with hospitalized ARI. A model including these variables plus cardiovascular disease (OR = 1.8, 95%CI: 0.9-3.5, p = 0.11) and pneumococcal vaccination (OR = 3.2, 95%CI: 0.9-11.5, p = 0.07) had a pseudo R^2^ of 0.28 (removal of the 2 latter variables led to a poorer fit (pseudo R^2^ = 0.20)).

**Table 3:** Comparison of ARI cases with matched hospital controls: analysis of vaccination status, underlying illness and other potential risk factors

			***ARI cases vs. matched hospital***			
**Variable**	**Cases (151)**		**Controls (246)**		***MOR (95% CI)***	***p***
	***N***	***%***	***N***	***%***		
**Pandemic influenza vaccination**	*150*	7	*246*	7	1.0 (0.4-2.5)	0.94
**Seasonal influenza vaccination 2009/10**	*150*	31	*237*	22	1.7 (1.1-2.8)	0.03
**Seasonal influenza vaccination 2008/09**	*144*	22	*239*	24	0.8 (0.4-1.5)	0.51
**Seasonal influenza vaccination 2007/08**	*142*	19	*239*	22	0.6 (0.4-1.7)	0.63
**Pneumococcal vaccine**	*131*	15	*211*	5	4.3 (1.5-11.8)	0.005
**Pandemic influenza vaccine offered by employer**	*148*	10	*245*	20	0.4 (0.2-0.8)	0.01
**Any chronic underlying illness**	*152*	67	*246*	57	4.5 (1.9-10.4	0.001
**Any chronic lung disease**	*151*	26	*246*	6	12.9 (4.5-36.4)	<0.0001
**COPD**	*150*	16	*246*	10	1.9 (1.0-3.6)	0.05
**Asthma**	*151*	16	*246*	10	2.1 (1.0-4.3)	0.05
**Chronic bronchitis**	*152*	35	*244*	18	3.4 (1.9-6.1)	<0.0001
**Cardiovascular disease**	*152*	39	*245*	30	1.9 (1.1-3.2)	0.02
**Immunosuppression**	*146*	15	*246*	0.4	35.2 (4.7-262.5)	0.001
**BMI ≥31**	*151*	21	*244*	24	0.9 (0.5-1.4)	0.54
**Frailty**	*151*	9	*246*	6	1.8 (0.8-4.0)	0.18
**≥ 3 physician visits in past year**	*151*	55	*242*	53	1.0 (0.7-1.6)	0.88
**≥ 1 hospitalization in past year**	*152*	30	*245*	34	0.9 (0.5-1.4)	0.57
**Current smoker**	*152*	44	*246*	47	0.9 (0.6-1.3)	0.53
**>20 packyears vs. non-smoker**	*152*	32	*246*	31	1.3 (0.7-2.3)	0.41
**Alcohol: ≥ 1 drink/week**	*150*	55	*234*	63	0.7 (0.4-1.0)	0.07
**Children in household**	*152*	38	*246*	19	2.7 (1.6-4.3)	<0.0001
**Public transport (> = 3x/week) vs. none**	*152*	41	*244*	44	0.9 (0.6-1.4)	0.52
**Owns car**	*152*	53	*243*	60	0.8 (0.5-1.1)	0.18
**Completion of at least Grade 10**	*149*	66	*239*	77	0.6 (0.4-0.9)	0.02
**Private health insurance**	*152*	15	*244*	19	0.6 (0.3-1.1)	0.11

### Vaccination status of HIV positive study participants

VE-analysis in subgroups with underlying illness was not feasible due to the small number of pH1N1-positive cases, but we examined the vaccination status of HIV positive study participants according to pH1N1 status (Table 4). Of 16 HIV-positive ARI with a definitive pH1N1 status, 2/3 (67%) pH1N1 positive patients and 4/13 (31%) pH1N1 negative patients were pH1N1 vaccinated (pχ2 = 0.62). Of the 3 HIV-positive hospitals controls with known pH1N1 vaccination status, 2 were pH1N1 vaccinated. As a group, HIV-positive study participants had a markedly higher pandemic (45%) as well as 2009/2010 seasonal influenza vaccination coverage (61%) than other study participants or the general population.

**Table 4:** pH1N1 vaccination status in HIV infected ARI cases and controls

	**HIV positive ARI cases**			**HIV positive hospital controls**	**Total**
**pH1N1 vaccination**	**pH1N1 positive**	**pH1N1 negative**	pH1N1 status unclear*		
Vaccinated**	2	4	2	2	10
**Not vaccinated**	1	9	1	1	12
**Unclear**	0	0	0	1	1
**Total**	3	13	3	4	23

*Swabbed late and did not provide serum samples.

**Single dose of Pandemrix 28–110 days prior to symptom onset with exception of one pH1N1-negative ARI case with 2 doses 119 and 91 days prior to symptom onset.

## Discussion

Despite the limited power of our study, the results suggest that AS03-adjuvanted pH1N1-vaccination implemented in Germany during the 2009 influenza pandemic was effective in preventing hospitalization with pH1N1 illness in adults ≤65 years of age. In another hospital-based case–control study using test-negative controls in Spain, a VE of 90% (95%CI: 48-100%) was found for hospitalization with pH1N1; however, only 12/34 vaccinated participants received Pandemrix® and 22 received non-adjuvanted vaccines [21]. In contrast, in a Danish register-based study of patients with underlying chronic illness, the VE estimate 14 days after Pandemrix® vaccination for the prevention of hospitalisation due to pH1N1-infection was 44% (95% CI-19-73%) [28]. In a number of studies in outpatients in general practice settings [23-26,28-32], VE estimates ranged from 49% to 100%, the lowest estimate being among older patients and those with underlying chronic conditions [28,30]. The only vaccine breakthroughs in our study were observed in 2/3 vaccinated HIV-positive patients, suggesting lower VE in this patient group. This is in line with the reported lower immunogenicity of the AS03-adjuvanted pH1N1-vaccine in HIV-infected persons [33,34].

### Limitations and strengths

The most serious limitation of our study was inadequate power. Due to the early onset and rapid decline of the pandemic wave, fewer patients than expected were included. The participation of 59% among hospitalized ARI-patients was also unexpectedly low, although recruited and non-recruited patients were similar regarding key demographic and clinical variables.

In addition, vaccination coverage among study participants was below our already pessimistic prognosis of 10%. This reflected the low coverage attained in the Berlin population of 6.8% (95%CI: 4.2–10.9%), only slightly better in persons with higher vaccination priority (12.8%; 95%CI: 11.4–14.4%) [7,35]. There was controversial public discussion in Germany, with 55% of participants in a representative telephone survey stating that information in the media led to doubt regarding the advisability of pH1N1-vaccination [11].

A further weakness was our inability to verify the vaccination status in all cases, as a vaccination registry was not available. Vaccination status remained undocumented in a high proportion of reportedly unvaccinated participants, however, this proportion was similar in cases, matched hospital and test-negative controls (Table 1). Several studies (reviewed in [36]) showed a high sensitivity (range 92–100), but lower specificity (38-100%) of self-reported influenza vaccination status in elderly persons. However, we suspect that the validity of recall of pandemic vaccination in adults ≤65 years may have been higher than for seasonal vaccination due to the extensive media coverage of the pandemic. Misclassification of vaccinated participants as unvaccinated among cases would lead to overestimation and among controls to underestimation of VE. In our study, any likelihood of misclassification appears similar in pH1N1 cases and their two control groups (Table 1).

Strengths of our study include the prospective recruitment of cases and controls, the use of two control groups, the exclusive use of a single pandemic vaccine, the use of serology to confirm pH1N1-infections that would otherwise not have been detected and the exclusion of controls with anamnestic or serological evidence of previous pH1N1-infection. Although we failed to obtain serum samples in relatively high proportion of ARI cases with negative (47/120; 39.2%) or – due to late testing – inconclusive (8/34; 23.5%) PCR results, results of patients who did provide sera suggest that a maximum of 1 patient each may have been wrongly classified as pH1N1-negative or as having an unclear pH1N1 status (Figure 1) when in fact pH1N1-positive: Of all 104 PCR negative patients with serum available, only 1 had positive serology (1%), and of the 26 patients with inconclusively negative PCR results due to late swabbing with serum available, 3 were seropositive (11.5%).

### Use of matched hospital and test-negative controls

Any control group should be representative of the population from which cases originate and have a risk of exposure to infection/vaccination similar to that of the cases [37-39]. Both our test-negative and community hospital controls originated from the same population as test-positive cases. Test-negative ARI patients had a similar risk of exposure to respiratory infections, presumably including pH1N1, as test-positive ARI cases. As we chose hospital controls with acute diagnoses or minor elective procedures, we assumed their potential risk of H1N1 exposure was also uninfluenced by their impending hospitalisation. In addition, we matched hospital controls according to date of admission and adjusted the test-negative analysis by period of influenza circulation. Furthermore, we matched hospital controls according to their vaccination probability based on official vaccination recommendations. Therefore, and because hospital controls, test-negative controls and H1N1 cases were similar with respect to frequency of smoking and social status, we believe that both control groups were appropriately selected for this study.

A comparison of the analyses using matched hospital controls and test-negative controls in this study is limited by the small numbers of recruited cases, vaccinated participants and lack of vaccination breakthroughs. The lower proportion of vaccinated controls in the test-negative comparison led to a lower point estimate and wider, non-significant 95%CI for the VE estimate than in the matched analysis. Besides vaccination probability based on official recommendations, an explanation for higher vaccination coverage of hospital controls could be more frequent use of out-patient health care resources. However, we found no significant difference regarding the proportion with any or >3 physician visits in the past year among hospital and test-negative controls, respectively (see Table 2). The use of pH1N1 test-negative ARI patients as controls has the practical advantage of eliminating the need for recruitment of additional controls, and has been widely used in VE studies performed in general practice sentinel networks [25,40]. The validity of this approach has been demonstrated, with a risk of underestimation of VE when the diagnostic test has low specificity or the ratio of influenza-confirmed to negative cases is low [41], as was the case in our study. On the other hand, an advantage of using hospital rather than test-negative controls is the ability to investigate potential risk factors for pH1N1-infection other than vaccination. While the comparison of pH1N1-positive cases with matched hospital and test-negative controls gave broadly similar results regarding pandemic vaccination, factors such as COPD, immunosuppression and contact to children in the household were only identified as potential risk factors for pH1N1-hospitalization when using matched hospital controls. This is presumably because such factors increase the risk of ARI regardless of aetiology, hence showing a similar distribution among test-positive and test-negative ARI cases and controls. Similarly, use of test-negative controls may result in inherent matching on factors potentially related to vaccination, leading to a more conservative estimate of protective effects, while, however, controlling for possible confounding factors.

### Seasonal influenza vaccination

We did not find evidence to suggest either increased or decreased risk of pH1N1-infections in participants vaccinated with current or past seasonal influenza vaccines, although the OR for seasonal vaccination in the two past seasons were non-significantly elevated in the test-negative comparison. Several Canadian studies using different study designs found an increased risk of pH1N1 illness during the initial pandemic wave in persons vaccinated with the seasonal 2008/2009 influenza vaccine [42,43]. Other studies showed no association [21,23,25,44-47] or a protective effect [48,49] of prior seasonal vaccination on pH1N1 illness. Besides possible biases or residual confounding, several hypotheses were put forward to explain a possibly true positive association [42,50-52].

### Risk factors identified for hospitalization with pH1N1/ARI

Of possible risk factors for pH1N1 hospitalization identified in our univariate matched analysis (Table 1), underlying respiratory illness, immunosuppression, and obesity have also been described by others as a risk factor for severe pH1N1-infection [53-56]. Having been offered influenza vaccination by one’s employer was inversely associated with pH1N1 hospitalization in univariate and with ARI in univariate and multivariate matched analysis. We hypothesize that such an offer may encourage higher awareness of infection risk or simply reflect a “healthy worker” effect. Car ownership was inversely and frequent use of public transport positively associated with hospitalization due to pH1N1 but not ARI in both the matched and the test-negative analyses. Car ownership was associated with serologically confirmed seasonal influenza in a cohort study of medical personnel, although only in persons from households without children [27]. We had too few pH1N1 cases to perform such a stratified analysis. Since we excluded controls with evidence of previous pH1N1-infection, acquired immunity through increased exposure in public transport settings cannot explain this observed inverse association. A “healthy commuter” effect may be an explanation; but we did not see such an association with ARI. The association of these two variables with mobility and with exposure to children in the household suggests that they were indirectly related to risk of pH1N1 exposure.

We did not find a significant association between various indicators of smoking and hospitalized pH1N1 illness or ARI, similar to many, but not all, other studies (reviewed in [57]). The proportion of smokers in the control groups (Table 2) was comparable to that in the Berlin population aged 15–64 years in 2009 at 43% [58]. Why previous hospitalization should be inversely associated with pH1N1-illness as observed in the matched analysis is not entirely clear. It may have been associated with a lower risk of pH1N1 exposure, e.g. through less social interaction.

Pneumococcal and seasonal 2009/2010, but not pH1N1 influenza vaccination were positively associated with ARI in univariate but not multivariate analysis. As shown by loss of statistical significance in multivariate analysis, this was due to the higher proportion of persons with an indication for these vaccines –such as COPD– among ARI patients than controls (“confounding by indication”). Contact to children in the household was a risk factor for hospitalization with ARI as well as pH1N1 illness, which is plausible as children have been shown to play an important role in the transmission of influenza and other viral respiratory illnesses to adults [59-62].

## Conclusions

Despite several study limitations, our results suggest that vaccination with the AS03-adjuvanted pH1N1-vaccine was effective at preventing hospitalizations due to pandemic influenza in adults, in keeping with the limited results seen in other hospital-based studies. To increase the reliability of VE estimates and to permit stratified analyses (e.g. VE for specific risk groups), results of studies assessing effectiveness of influenza vaccines for prevention of severe disease from different centres could be pooled to increase power, as currently performed within I-MOVE (Influenza Monitoring Vaccine Effectiveness in Europe) for the assessment of influenza VE in outpatient sentinels [25]. The use of hospital but not test-negative controls showed a statistically protective effect of pH1N1-vaccination and permitted the integrated assessment of risk factors for pH1N1-infection. Further studies are warranted that compare the use and impact of different control groups when assessing influenza VE in post-marketing studies.

## Competing interests

The authors declare that they have no competing interests.

## Authors’ contributions

WH, OW and CT participated in designing the study. WH, GH, MN, PJ and BG participated in data collection and analysis. BS performed PCR and serological influenza diagnosis. WH drafted the manuscript. All authors participated in interpretation of the data, critically reviewed and approved the final version of the manuscript.

## References

[B1] Chan M World now at the start of 2009 influenza pandemic 2009 WHO, Geneva http://www.who.int/mediacentre/news/statements/2009/h1n1_pandemic_phase6_20090611/en/index.html. 2009. 21-9-2009

[B2] Perez-Padilla R de la Rosa-Zamboni D Ponce de Leon S Hernandez M Quinones-Falconi F Bautista E Ramirez-Venegas A Rojas-Serrano J Ormsby CE Corrales A Pneumonia and respiratory failure from swine-origin influenza A (H1N1) in Mexico N Engl J Med 2009 361 680 689 19564631 10.1056/NEJMoa0904252

[B3] Poggensee G Gilsdorf A Buda S Eckmanns T Claus H Altmann D Krause G Haas W RKI Working Group The first wave of pandemic influenza (H1N1) 2009 in Germany: from initiation to acceleration BMC Infect Dis 2010 10 155 10.1186/1471-2334-10-155 20525408 PMC2902478

[B4] Roman F Vaman T Gerlach B Markendorf A Gillard P Devaster JM Immunogenicity and safety in adults of one dose of influenza A H1N1v 2009 vaccine formulated with and without AS03A-adjuvant: preliminary report of an observer-blind, randomised trial Vaccine 2010 28 1740 1745 20034605 10.1016/j.vaccine.2009.12.014

[B5] Roman F Vaman T Kafeja F Hanon E Van Damme P AS03A-adjuvanted influenza A (H1N1) 2009 vaccine for adults up to 85 years of age Clin Infect Dis 2010 51 668 677 20687838 10.1086/655830

[B6] Gilsdorf A Poggensee G Working group pandemic influenza A(H1N1)v: influenza A(H1N1)v in Germany: the first 10,000 cases Euro Surveill 2009 14 1 4 10.2807/ese.14.34.19318-en 19712649

[B7] Walter D Heiden Mad Reiter S Krause G Wichmann O Monitoring pandemic influenza A(H1N1) vaccination coverage in Germany 2009/10 - results from thirteen consecutive cross-sectional surveys Vaccine 2011 29 4008 4012 21463683 10.1016/j.vaccine.2011.03.069

[B8] Gesundheit Senatsverwaltung für Umwelt Verbraucherschutz und Ergänzende FAQs zum verwendeten Impfstoff und zum Ablauf der Impfung gegen Influenza A/H1N1 in Berlin (Stand: 16.10.2009) [Additional FAQs regarding the available vaccine and procedures for vaccinating against Influenza A/H1N1 in Berlin (Dated: 16.10.2009)] 2009 Senatsverwaltung für Gesundheit, Verbraucherschutz und Umwelt, Berlin http://www.berlin.de/imperia/md/content/sen-gesundheit/notfallvorsorge/pandemie/impfen/erg__nzende_faqs_b__rger.pdf?start&ts=1268747044&file=erg__nzende_faqs_b__rger.pdf

[B9] Demicheli V Di PC Jefferson T Rivetti A Rivetti D Vaccines for preventing influenza in healthy adults Cochrane Database Syst Rev 2007 2 CD001269 17443504 10.1002/14651858.CD001269.pub3

[B10] Jefferson T DiPietrantonj C Rivetti A Bawazeer GA Al-Ansary LA Ferroni E Vaccines for preventing influenza in the elderly (Review) Cochrane Database Syst Rev 2010 7 CD004876 20166072 10.1002/14651858.CD004876.pub3

[B11] Koch-Institut R Telefonische Erhebung zur Impfung gegen die pandemische Influenza (H1N1) 2009 [Telephone survey regarding vaccination against pandemic influenza (H1N1) 2009] Epidemiol Bull 2010 13 114 115

[B12] Schulze M Nitsche A Schweiger B Biere B Diagnostic approach for the differentiation of the pandemic influenza A(H1N1)v virus from recent human influenza viruses by real-time PCR PLoS One 2010 5 e9966 20376359 10.1371/journal.pone.0009966 PMC2848602

[B13] Biere B Bauer B Schweiger B Differentiation of influenza B virus lineages yamagata and victoria by real-time PCR J Clin Microbiol 2010 48 1425 1427 20107085 10.1128/JCM.02116-09 PMC2849545

[B14] Rowe T Abernathy RA Hu-Primmer J Thompson WW Lu X Lim W Fukuda K Cox NJ Katz JM Detection of antibody to avian influenza A (H5N1) virus in human serum by using a combination of serologic assays J Clin Microbiol 1999 37 937 943 10074505 10.1128/jcm.37.4.937-943.1999 PMC88628

[B15] Cowling BJ Chan KH Fang VJ Lau LLH So HC Fung ROP Ma ESK Kwong ASK Chan CW Tsui WWS Comparative epidemiology of pandemic and seasonal influenza A in households N Engl J Med 2010 362 2175 2184 20558368 10.1056/NEJMoa0911530 PMC4070281

[B16] De Serres G Rouleau I Hamelin M-È Quach C Skworonski D Flamand L Boulianne N Li Y Carbonneau J Bourgault A-M Contagious period for pandemic (H1N1) 2009 Emerg Infect Dis 2010 16 783 788 20409367 10.3201/eid1605.091894 PMC2954014

[B17] Suess T Buchholz U Dupke S Grunow R Matthias an dH Heider A Biere B Schweiger B Haas W Krause G Shedding and transmission of novel influenza virus A/H1N1 infection in households--Germany, 2009 Am J Epidemiol 2010 171 1157 1164 20439308 10.1093/aje/kwq071

[B18] Lee N Chan PK Hui D Rainer TH Wong E Choi K-W Lui GC Wong BC Wong RY Lam W-Y Viral loads and duration of viral shedding in adult patients hospitalized with influenza J Infect Dis 2009 200 492 500 19591575 10.1086/600383 PMC7110250

[B19] Leekha S Zitterkopf NL Espy MJ Smith TF Thompson RL Sampathkumar P Duration of Influenza A shedding in hospitalized patients and implications for infection control Infect Control Hosp Epidemiol 2007 28 1071 1076 17932829 10.1086/520101

[B20] Arbeitsgemeinschaft Influenza Bericht zur Epidemiologie der Influenza in Deutschland Saison 2009/10 2011 Robert Koch-Institute, Berlin

[B21] Puig-Barberà J Arnedo-Pena A Pardo-Serrano F Tirado-Balaguer MD Pérez-Vilar S Silvestre-Silvestre E Calvo-Mas C Safont-Adsuara L Ruiz-García M Effectiveness of seasonal 2008–2009, 2009–2010 and pandemic vaccines, to prevent influenza hospitalizations during the autumn 2009 influenza pandemic wave in Castellón, Spain. A test-negative, hospital-based, case–control study Vaccine 2010 28 7460 7467 20875486 10.1016/j.vaccine.2010.09.042

[B22] Nicholson KG Abrams KR Batham S Clark TW Hoschler K Lim WS Medina MJ Nguyen-Van-Tam JS Read RC Warren FC Immunogenicity and safety of a two-dose schedule of whole-virion and AS03A-adjuvanted 2009 influenza A (H1N1) vaccines: a randomised, multicentre, age-stratified, head-to-head trial Lancet Infect Dis 2011 11 91 101 21168369 10.1016/S1473-3099(10)70296-6

[B23] Hardelid P Fleming D McMenamin J Andrews N Robertson C Sebastianpillai P Ellis J Carman W Wreghitt T Watson J Effectiveness of pandemic and seasonal influenza vaccine in preventing pandemic influenza A(H1N1)2009 infection in England and Scotland 2009–2010 Euro Surveill 2011 16 2 pii=19763 Available online: http://www.eurosurveillance.org/ViewArticle.aspx?ArticleId=19763 21251487

[B24] Van Buynder PG Dhaliwal JK Van Buynder JL Couturier C Minville-Leblanc M Garceau R Tremblay FW Protective effect of single-dose adjuvanted pandemic influenza vaccine in children Influenza Other Respi Viruses 2010 4 171 178 20629771 10.1111/j.1750-2659.2010.00146.x PMC5964543

[B25] Valenciano M Kissling E Cohen JM Oroszi B Barret AS Rizzo C Nunes B Pitigoi D Larrauri CÃ¡mara A Mosnier A Estimates of pandemic influenza vaccine effectiveness in Europe, 2009–2010: results of influenza monitoring vaccine effectiveness in Europe (I-MOVE) multicentre case–control study PLoS Med 2011 8 e1000388 21379316 10.1371/journal.pmed.1000388 PMC3019108

[B26] Simpson CR Ritchie LD Robertson C Sheikh A McMenamin J Vaccine effectiveness in pandemic influenza – primary care reporting (VIPER): an observational study to assess the effectiveness of the pandemic influenza A (H1N1)v vaccine Health Technol Assess 2010 14 313 346 20630126 10.3310/hta14340-05

[B27] Williams C Schweiger B Diner G Gerlach F Haaman F Krause G Nienhaus A Buchholz U Seasonal influenza risk in hospital healthcare workers is more strongly associated with household than occupational exposures: results from a prospective cohort study in Berlin, Germany, 2006/07 BMC Infect Dis 2010 10 8 20067628 10.1186/1471-2334-10-8 PMC2836320

[B28] Emborg H-D Krause TG Hviid A Simonsen J Molbak K Effectiveness of vaccine against pandemic influenza A/H1N1 among people with underlying chronic diseases: cohort study, Denmark, 2009–10 BMJ 2012 344 d7901 22277542 10.1136/bmj.d7901

[B29] Skowronski DM Janjua NZ De Serres G Hottes TS Dickinson JA Crowcroft N Kwindt TL Tang P Charest H Fonseca K Effectiveness of AS03 adjuvanted pandemic H1N1 vaccine: case–control evaluation based on sentinel surveillance system in Canada, autumn 2009 BMJ 2011 342 c7297 21292718 10.1136/bmj.c7297 PMC3033439

[B30] Andrews N Waight P Yung CF Miller E Age-specific effectiveness of an oil-in-water adjuvanted pandemic (H1N1) 2009 vaccine against confirmed infection in high risk groups in England J Infect Dis 2011 203 32 39 21148494 10.1093/infdis/jiq014 PMC3086445

[B31] Ortqvist A Berggren I Insulander M de Jong B Svenungsson B Effectiveness of an adjuvanted monovalent vaccine against the 2009 pandemic strain of influenza a(H1N1)v in stockholm county, Sweden Clin Infect Dis 2011 52 1203 1211 21507917 10.1093/cid/cir182

[B32] Castilla J Morán J Martinez-Artola V Fernández-Alonso M Guevara M Cenoz MG Reina G Alvarez N Arriazu M Elía F Effectiveness of the monovalent influenza A(H1N1)2009 vaccine in Navarre, Spain, 2009–2010: cohort and case–control study Vaccine 2011 29 5919 5924 21723358 10.1016/j.vaccine.2011.06.063

[B33] Bickel M Wieters I Khaykin P Nisius G Haberl A Stephan C Von HN Herrmann E Doerr HW Brodt HR Low rate of seroconversion after vaccination with a split virion, adjuvanted pandemic H1N1 influenza vaccine in HIV-1-infected patients AIDS 2010 24 F31 F35 20559034 10.1097/QAD.0b013e3283398da1

[B34] Tremblay CL Rouleau D Fortin C Toma E Sylla M Cyr L Cote S Baz M Sampalis J Trautman L Immunogenicity and tolerability of an inactivated and adjuvanted pandemic H1N1 influenza vaccine, in HIV-1-infected patients Vaccine 2011 29 1359 1363 21185423 10.1016/j.vaccine.2010.12.023

[B35] Robert Koch-Institut Repräsentative telefonische Erhebung zur Impfung gegen die pandemische Influenza (H1N1) 2009. Ergebnisse aus Befragungen bis April 2010 [Representative telephone survey on vaccination against pandemic influenza (H1N1) 2009. Results to April 2010] Epidemiol Bull 2010 25 237 238

[B36] Skull SA Andrews RM Byrnes GB Kelly HA Nolan TM Brown GV Campbell DA Validity of self-reported influenza and pneumococcal vaccination status among a cohort of hospitalized elderly inpatients Vaccine 2007 25 4775 4783 17499402 10.1016/j.vaccine.2007.04.015

[B37] Grimes DA Schulz KF Compared to what? Finding controls for case–control studies Lancet 2005 365 1429 1433 15836892 10.1016/S0140-6736(05)66379-9

[B38] Wacholder S McLaughlin JK Silverman DK Mandel JS Selection of controls in case–control studies I.Principles Am J Epidemiol 1992 135 1019 1028 1595688 10.1093/oxfordjournals.aje.a116396

[B39] Wacholder S Silverman DK McLaughlin JK Mandel JS Selection of Controls in Case–control Studies II. Types of Controls Am J Epidemiol 1992 135 1029 1041 1595689 10.1093/oxfordjournals.aje.a116397

[B40] Valenciano M Kissling E Ciancio BC Moren A Study designs for timely estimation of influenza vaccine effectiveness using European sentinel practitioner networks Vaccine 2010 28 7381 7388 20851086 10.1016/j.vaccine.2010.09.010

[B41] Orenstein EW De Serres G Haber MJ Shay DK Bridges CB Gargiullo P Orenstein WA Methodologic issues regarding the use of three observational study designs to assess influenza vaccine effectiveness Int J Epidemiol 2007 36 623 631 17403908 10.1093/ije/dym021

[B42] Skowronski DM De SG Crowcroft NS Janjua NZ Boulianne N Hottes TS Rosella LC Dickinson JA Gilca R Sethi P Association between the 2008–09 seasonal influenza vaccine and pandemic H1N1 illness during Spring-Summer 2009: four observational studies from Canada PLoS Med 2010 7 e1000258 20386731 10.1371/journal.pmed.1000258 PMC2850386

[B43] Janjua NZ Skowronski DM Hottes TS Osei W Adams E Petric M Sabaiduc S Chan T Mak A Lem M Seasonal influenza vaccine and increased risk of pandemic A/H1N1-related illness: first detection of the association in British Columbia, Canada Clin Infect Dis 2010 51 1017 1027 20887210 10.1086/656586 PMC7107856

[B44] Carcione DP Giele C Goggin LS Kwan KS Smith DW Dowse GK Mak DB Effler P Association between 2009 seasonal influenza vaccine and influenza-like illness during the 2009 pandemic: preliminary results of a large household transmission study in Western Australia Euro Surveill 2010 15 28 pii=19616 Available online: http://www.eurosurveillance.org/ViewArticle.aspx?ArticleId=19616 20650055

[B45] Iuliano AD Reed C Guh A Desai M Dee DL Kutty P Gould LH Sotir M Grant G Lynch M Notes from the field: outbreak of 2009 pandemic influenza a (H1N1) virus at a large public university in Delaware, April-May 2009 Clin Infect Dis 2009 49 1811 1820 19911964 10.1086/649555

[B46] Kelly HA Grant KA Fielding JE Carville KS Looker CO Tran T Jacoby P Pandemic influenza H1N1 2009 infection in Victoria, Australia: no evidence for harm or benefit following receipt of seasonal influenza vaccine in 2009 Vaccine 2011 29 6419 6426 21473950 10.1016/j.vaccine.2011.03.055

[B47] Pebody R Andrews N Waight P Malkani R McCartney C Ellis J Miller E No effect of 2008/09 seasonal influenza vaccination on the risk of pandemic H1N1 2009 influenza infection in England Vaccine 2011 29 2613 2618 21292008 10.1016/j.vaccine.2011.01.046

[B48] Echevarría-Zuno S Mejía-Aranguré JM Mar-Obeso AJ Grajales-Muniz C Robles-Pérez E González-León M Ortega-Alvarez MC Gonzalez-Bonilla C Rascón-Pacheco RA Borja-Aburto VH Infection and death from influenza A H1N1 virus in Mexico: a retrospective analysis Lancet 2009 374 2072 2079 19913290 10.1016/S0140-6736(09)61638-X

[B49] Garcia-Garcia L Valdespino-Gomez JL Lazcano-Ponce E Jimenez-Corona A Higuera-Iglesias A Cruz-Hervert P Cano-Arellano B Garcia-Anaya A Ferreira-Guerrero E Baez-Saldana R Partial protection of seasonal trivalent inactivated vaccine against novel pandemic influenza A/H1N1 2009: case–control study in Mexico City BMJ 2009 339 b3928 19808768 10.1136/bmj.b3928 PMC2758337

[B50] Glezen WP How did the 2008−2009 seasonal influenza vaccine affect the pandemic? Clin Infect Dis 2010 51 1380 1382 21067354 10.1086/657312

[B51] Kelly H Barry S Laurie K Mercer G Seasonal influenza vaccination and the risk of infection with pandemic influenza: a possible illustration of non-specific temporary immunity following infection Euro Surveill 2010 15 47 pii=19722 Available online: http://www.eurosurveillance.org/ViewArticle.aspx?ArticleId=19722 10.2807/ese.15.47.19722-en 21144441

[B52] Mercer G Kelly H Seasonal influenza vaccination and the 2009 pandemic Clin Infect Dis 2011 52 828 829 21367738 10.1093/cid/cir035

[B53] Buda S Köpke K Haas W Epidemiologischer Steckbrief der pandemischen Influenza (H1N1) 2009 basierend auf Einzelfallmeldungen nach Infektionsschutzgesetz Bundesgesundheitsbl - Gesundheitsforsch - Gesundheitsschutz 2010 53 1223 1230 10.1007/s00103-010-1158-0 21161471

[B54] Jain S Kamimoto L Bramley AM Schmitz AM Benoit SR Louie J Sugerman DE Druckenmiller JK Ritger KA Chugh R Hospitalized patients with 2009 H1N1 influenza in the United States, April-June 2009 N Engl J Med 2009 361 1935 1944 19815859 10.1056/NEJMoa0906695

[B55] Morgan OW Bramley A Fowlkes A Freedman DS Taylor TH Gargiullo P Belay B Jain S Cox C Kamimoto L Morbid Obesity as a Risk Factor for Hospitalization and Death Due to 2009 Pandemic Influenza A(H1N1) Disease PLoS One 2010 5 e9694 20300571 10.1371/journal.pone.0009694 PMC2837749

[B56] Louie JK Acosta M Samuel MC Schechter R Vugia DJ Harriman K Matyas BT the CP A novel risk factor for a novel virus: obesity and 2009 pandemic influenza A (H1N1) Clin Infect Dis 2011 52 1 12 21208911 10.1093/cid/ciq152

[B57] Murin S Bilello KS Respiratory Tract Infections: another reason not to smoke Cleve Clin J Med 2005 72 916 920 16231688 10.3949/ccjm.72.10.916

[B58] Federal Statistical Office Mikrozensus - Fragen zur Gesundheit - Rauchgewohnheiten der Bevölkerung 2009 [Microcensus - Health Questions - Smoking habits in the German population 2009] 2009 the Federal Statistical Office, Mainz http://www.destatis.de/jetspeed/portal/cms/Sites/destatis/Internet/DE/Content/Publikationen/Fachveroeffentlichungen/Gesundheit/Gesundheitszustand/Rauchgewohnheiten5239004099004,property=file.pdf

[B59] Brownstein JS Mandl KD Pediatric population size is associated with geographic patterns of Acute Respiratory Infections among adults Ann Emerg Med 2008 52 63 68 18374453 10.1016/j.annemergmed.2008.02.009 PMC2597284

[B60] Brownstein JS Kleinman KP Mandl KD Identifying pediatric age groups for influenza vaccination using a real-time regional surveillance system Am J Epidemiol 2005 162 686 693 16107568 10.1093/aje/kwi257 PMC1266301

[B61] Musher DM How contagious are common respiratory tract infections? N Engl J Med 2003 348 1256 1266 12660390 10.1056/NEJMra021771

[B62] Viboud C Boelle P Cauchemez S Lavenu A Valleron A Antoine F Carrat F Risk factors of influenza transmission in households Br J Gen Pract 2004 54 684 689 15353055 PMC1326070

